# A Novel Classification Method for a Driver’s Cognitive Stress Level by Transferring Interbeat Intervals of the ECG Signal to Pictures

**DOI:** 10.3390/s20051340

**Published:** 2020-02-29

**Authors:** Jing Huang, Xiong Luo, Xiaoyan Peng

**Affiliations:** Research Centre of Vehicle and Traffic Safety, State Key Laboratory of Advanced Design and Manufacturing for Vehicle Body, Hunan University, Changsha 410082, China; xiong_luo@hnu.edu.cn (X.L.); xiaoyan_p@126.com (X.P.)

**Keywords:** traffic safety, cognitive stress level, convolution neural network, BP neural network, ECG signal

## Abstract

In this study, a novel classification method for a driver’s cognitive stress level was proposed, whereby the interbeat intervals extracted from an electrocardiogram (ECG) signal were transferred to pictures, and a convolution neural network (CNN) was used to train the pictures to classify a driver’s cognitive stress level. First, we defined three levels of tasks and collected the ECG signal of the driver at different cognitive stress levels by designing and performing a driving simulation experiment. We extracted the interbeat intervals and converted them to pictures according to the number of consecutive interbeat intervals in each picture. Second, the CNN model was used to train the data set to recognize the cognitive stress levels. Classification accuracies of 100%, 91.6% and 92.8% were obtained for the training set, validation set and test set, respectively, and were compared with those the BP neural network. Last, we discussed the influence of the number of interbeat intervals in each picture on the performance of the proposed classification method. The results showed that the performance initially improved with an increase in the number of interbeat intervals. A downward trend was observed when the number exceeded 40, and when the number was 40, the model performed best with the highest accuracy (98.79%) and a relatively low relative standard deviation (0.019).

## 1. Introduction

Traffic safety has always been a critical global problem. For both highway traffic and urban traffic, the influencing factors of traffic accidents, including human factors, vehicle factors and road factors, are complicated, and a driver has the most important role in the human-vehicle-road environment. In 2018, the National Highway Traffic Safety Administration (NHTSA) reported that 3,157 fatal crashes occurred on U.S. roadways in 2016, of which 3450 fatalities involved distracted drivers [1]. With the development of advanced driving assistant systems and automatic driving, monitoring a driver’s cognitive status in real time is more meaningful. On the one hand, monitoring of a driver’s cognitive stress level is needed to develop the warning function or automatic operation correction function of a driving assistant system; on the other hand, when the driving rights of the automatic driving need to be handed over to the driver, knowledge of a driver’s cognitive status is necessary.

During driving, drivers often perform actions that are unrelated to the driving task, such as making telephone calls, talking with passengers in the car, listening to music, smoking, eating, and thinking about matters unrelated to driving. These behaviors may decrease drivers’ driving ability and increase the risk of accidents. With the development of technology and an increase in in-vehicle electronic devices, the number of sources that distract drivers has increased. We refer to tasks that are unrelated to the driving task as secondary tasks. Ratcliff et al. used a one-boundary diffusion model to model the data from two experiments, in which subjects performed a simple simulated driving task and observed that drivers would take longer to react in response to an unexpected situation when performing a secondary task, such as taking a mobile call [2]. Some researchers discovered that drivers will pay attention to the vehicle in front of them but disregard the instrument panel, side mirrors, and surrounding objects due to the increase in workload caused by an in-vehicle secondary task [3,4]. Strayer et al. noted that a driver’s ability to scan, predict, identify, make decisions, and execute a response was affected when driving while performing an unrelated secondary task [5]. These reactions will affect a driver’s operation and increase the likelihood of a car accident. According to the NHTSA, distracting driving can be categorized as three types: visual distraction, manual distraction and cognitive distraction [6].

Recently, an increasing number of researchers have become committed to studying the monitoring and classification methods of driver status, which can be divided into three aspects according to the research objects: (1) classification methods based on the driving data (vehicle speed, vehicle acceleration, steering wheel angle, etc.); (2) classification methods based on drivers’ behaviors acquired by a camera; and (3) classification methods based on drivers’ physical data. For the first type, Chen et al. observed a significant difference in the lateral acceleration rate and yaw rate between “normal driving” and drowsy/distracted driving. Their study also showed that the lateral acceleration rate and yaw rate during drowsy/distracted driving were significantly larger than those during normal driving [7]. Based on a driver’s driving data, this type of method will be affected by the driver’s experience, the vehicle type and the road condition, which can interfere with detection and increase the difficulty of accurate classification. For the second type, Abouelnaga et al. proposed a novel system that consists of a genetically weighted ensemble of convolutional neural networks (CNNs) and achieved a driving posture estimation classification accuracy of 95.98% [8]. Mbouna et al. applied a support vector machine (SVM) to classify whether the driver in a driving event is alert or not alert based on the eye state and head pose and accomplished continuous monitoring of the alertness of a vehicle driver [9]. Craye et al. utilized drivers’ behavior features (eye behavior, arm position, head orientation, and facial expressions) to classify a driver’s distracted state and obtained a 90% classification accuracy [10]. However, these methods of obtaining information via a camera may involve privacy issues and have a high demand on the ambient light. Some special cognitive distraction circumstances, such as “looked but failed to see”, are not suitable for this type of method. For the third type, Tjolleng et al. applied an artificial neural network based on the ECG signal to classify a driver’s cognitive workload and achieved an 82% classification accuracy [11]. Research conducted by Guardiola et al. revealed an increase in heart anomalies during high-stress driving, which can serve as an index to weigh a driver’s workload [12]. This type of method is reliable because the physiological data can directly reflect a driver’s physiological state and will change when the driver’s attention changes, and the process of signal acquisition is more convenient and easier with the development of wireless signal acquisition technology. Researchers in this field are paying more attention to the application of physiological signals, such as electrocardiogram (ECG) [13,14,15], electroencephalogram (EEG) [16,17,18,19,20,21], galvanic skin response (GSR) [22,23,24,25], and electrooculogram (EOG) [26,27,28]. Improving the accuracy and reliability of recognition is a research goal of scholars.

In this study, a novel method for classifying a driver’s cognitive stress level by transferring interbeat intervals of an ECG signal to pictures was proposed, and the classification accuracy of the proposed method was compared with that of the common BP neural network classification method.

## 2. Materials and Methods

### 2.1. Novel Classification Method for a Driver’s Cognitive Stress Level

Although traditional machine learning methods can achieve a reasonable effect in the detection of drivers’ statuses, they have some limitations. Constructing a pattern-recognition or machine learning system requires careful engineering and considerable domain expertise to design a feature extractor that transforms raw data (such as original ECG signals) into a suitable internal representation or feature vector from which the learning subsystem, often a classifier, can detect or classify patterns in the input [29]. In this process, the selection of features is very important and has a considerable impact on the accuracy of the final detection or classification. Identifying the most suitable features for the model is difficult and time consuming.

In this paper, we proposed a novel method based on deep learning to detect and classify drivers’ cognitive stress levels. Because traditional machine learning methods require a substantial amount of time to extract features from data and we may not be able to manually obtain the best features, we employ a deep learning algorithm. Deep learning methods do not require feature engineering, which means that we can achieve satisfactory performance by inputting raw data into a deep learning network. A CNN is a kind of feedforward neural network with convolutional computation and a deep structure, and is a representative deep learning algorithm. A convolutional neural network is constructed according to the visual perception mechanism of a creature, which can perform supervised learning and unsupervised learning. The convolutional kernel parameter sharing and sparseness of the interlayer connection in the hidden layer enable the convolutional neural network to learn grid-like topology features, such as pixels and audio, with less calculation and have a stable effect and no additional feature engineering requirements for the data. We intend to apply a CNN model; thus, we need to make some changes to our data [30,31]. 

**Figure 1:**
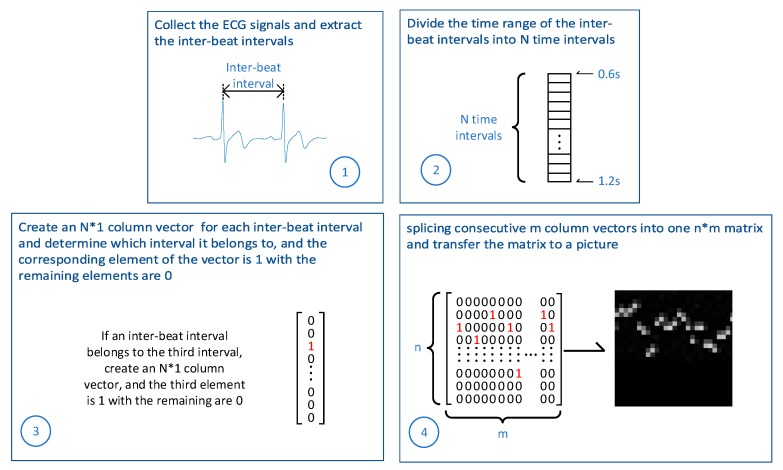
A novel classification method for a driver’s cognitive stress level.

As shown in Figure 1, first, we collect the ECG signals of a driver’s different statuses by simulation experiments and extract the interbeat intervals from the ECG signals. Second, we divide the entire time interval of the interbeat intervals into several intervals according to the distribution of the interbeat intervals. The number of divisions is N. Third, we create an N × 1 column vector for each interbeat interval and determine to which interval the interbeat belongs. The corresponding element of the vector is 1, and the remaining elements are 0. Fourth, we splice consecutive m column vectors into one n × m matrix and transfer the matrix to a picture. Last, we use these pictures as the data set for subsequent training.

### 2.2. Data Source

**Figure 2:**
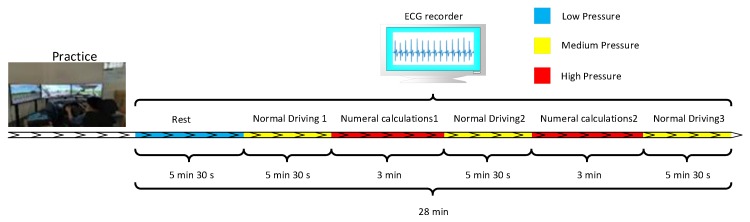
Driving simulation experiment.

To obtain the data to verify the feasibility of the proposed method, we have designed and performed a simulation experiment to simulate the different stress levels of a driver. We set numeral calculations as the cognitive distracting task, and the calculation difficulty is a two-digit addition and subtraction method that requires carrying and borrowing. The participants were instructed to drive a driving simulator (primary task) while performing a numeral calculation task (secondary task). In this paper, we define the rest state as a low cognitive stress level (as shown in the blue phase of Figure 2), the normal driving state as a normal cognitive stress level (as shown in the yellow phase of Figure 2) and the distracted driving state as a high cognitive stress level (as shown in the red phase of Figure 2). All subjects gave their informed consent for inclusion before they participated in the study. The study was conducted in accordance with the Declaration of Helsinki, and the protocol was approved by the Second Xiangya Hospital of Central South University Institutional Review Board.

The entire experiment was divided into seven sections: Practice, Rest (lasting 5 min and 30 s), Normal driving 1 (lasting 5 min and 30 s), Numeral calculations 1 (lasting 3 min), Normal driving 2 (lasting 5 min and 30 s), Numeral calculations 2 (lasting 3 min), and Normal driving 3 (lasting 5 min and 30 s). The ECG data, with the exception of the practice phase, was recorded. As shown in Figure 2, the practice phase was to familiarize the driver with the driving simulator; the collection of ECG data in this phase was not required. In the rest state, data were collected when the driver was at rest. In the normal driving period, data was collected when the driver was under normal pressure. In the numeral calculation period, a high-pressure state of the driver was simulated, and the driver’s ECG data were collected.

Considering the security issues, the entire experiment was performed using the scientific vehicle driving simulator produced by FORUM8 Co., Ltd. (Tokyo, Japan), and the map modeling software was the UC-win/Road driving simulation software. The ECG signal was recorded by the MP150-BioNomadix multichannel wireless physiological analysis recorder (BIOPAC Systems Inc., Goleta, CA, USA).

### 2.3. Signal Preprocessing

**Figure 3:**
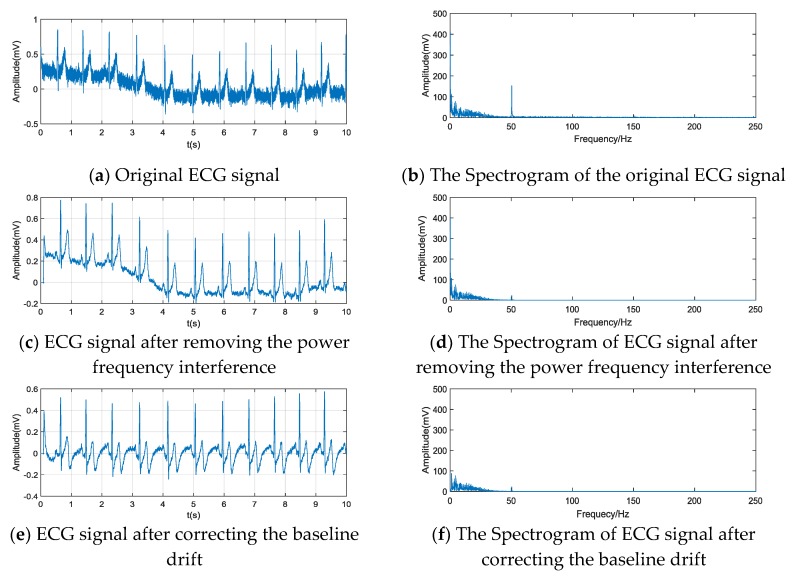
ECG signals before and after filtering and their spectrograms.

Noise is inevitable when collecting ECG data, and the main sources of noise are the power frequency interference and the baseline drift. To accurately extract the relevant information in the ECG signal, we need to remove the noise interference in the ECG signal before extraction. The power frequency interference is a noise problem caused by electromagnetic radiation in the surrounding environment during the operation of the equipment; its frequency is 50 Hz. The baseline drift refers to the instability of the working conditions of the instrument when measuring the ECG signal, leading to the baseline of the ECG signal being not a horizontal line but oscillating up and down. As shown in Figure 3a,b, two main sources of noise exist in the signal. The effects of these noises can be substantially removed by different digital filtering methods. Recently, the Blind Source Separation (BSS) techniques such as Independent Component Analysis (ICA) are wildly used for the artifact removal of the physiological signals [32,33,34], however, our signals are single channel signals while the BSS techniques requires multi-channels signals, thus, we choose the traditional methods to remove the noise. First, we applied a notch filter to remove the effect of the power frequency interference. As we can see from the ECG signal after removing the power frequency interference, shown in Figure 3c, and its spectrogram, shown in Figure 3d, the majority of the 50 Hz noise was removed. We utilized a zero-phase filter to correct the baseline drift; the effect is shown in Figure 3e,f.

### 2.4. Data Set for the Proposed Novel Method with a CNN Model

**Figure 4:**
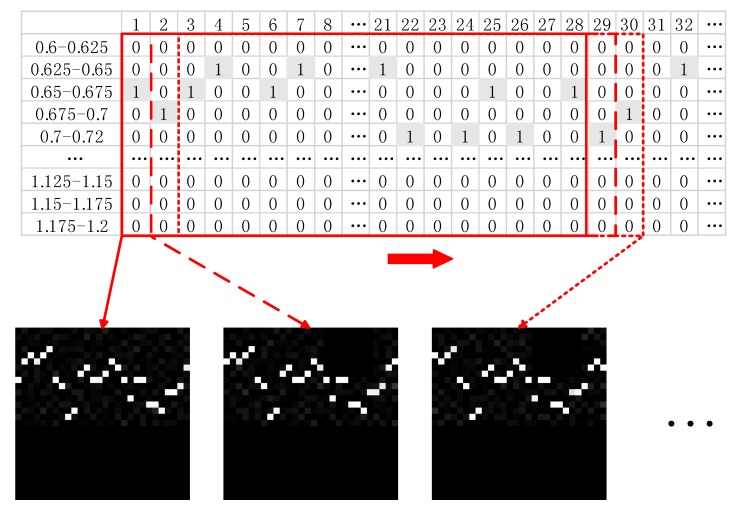
Data sets for the CNN model (number of interbeat intervals is 28).

After denoising the ECG signal, we can start to construct the data set. First, for the ECG signal of the rest phase and normal driving 1 phase, consider the middle five minutes as the data for the low pressure and medium pressure states, and separately consider the middle 2.5 min of the ECG signal of numeral calculations 1 and numeral calculations 2 as the data for the high pressure state, which is done to avoid the transition effects of different state transitions. Second, use the pan_tompkin algorithm [35] coded in MATLAB to acquire the IBI data, and remove the abnormal points outside the normal range of IBIs (0.6 s–1.2 s). Third, divide the IBI data into 28 intervals from 0.6 s to 1.2 s as follows: [0.6, 0.625), [0.625, 0.65), [0.65, 0.675), [0.675, 0.7), [0.7, 0.72), [0.72, 0.74), [0.74, 0.76), [0.76, 0.78), [0.78, 0.8), [0.8, 0.82), [0.82, 0.84), [0.84, 0.86), [0.86, 0.88), [0.88, 0.9), [0.9, 0.92), [0.92, 0.94), [0.94, 0.96), [0.96, 0.98), [0.98, 1), [1, 1.02), [1.02, 1.04), [1.04, 1.06), [1.06, 1.08), [1.08, 1.1), [1.1, 1.125), [1.125, 1.15), [1.15, 1.175), and [1.175, 1.2]. Fourth, determine to which interval each IBI belongs to obtain the matrix shown in Figure 4. Last, convert the 28 consecutive IBIs to pictures to obtain the data sets for the CNN model (as shown in the red box; the number 28 is randomly selected, which will be discussed later).

### 2.5. CNN Modeling

**Figure 5:**
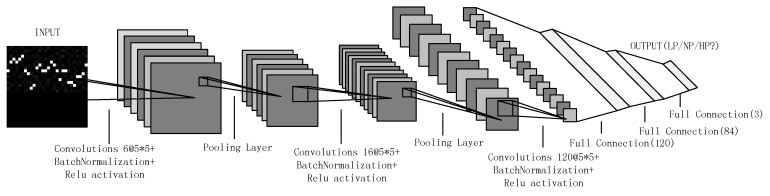
Structure of the CNN model.

In this research, we build an 8-layer CNN model; the CNN structure is illustrated in Figure 5. The first five layers are convolution layers and pooling layers, and the pooling layer is positioned between two convolution layers. Each convolution layer includes 6 filters, 16 filters and 120 filters, and each filter is a convolution kernel of a 5 × 5 receptive field. The size of the pooling layers is 2 × 2. The last several layers are fully connected layers, and their depths are 120, 84 and 3. The input of the CNN model is the previously acquired data set. We divided the data set into three parts: the training set, the validation set, and the test set. The first 70% of the data in chronological order were the training set, and the remaining data were randomly divided into two equal-number data sets for the validation set and the test set. The outputs were low cognitive pressure (LP), normal cognitive pressure (NP), and high cognitive pressure (HP).

### 2.6. Comparative Experiment

**Figure 6:**
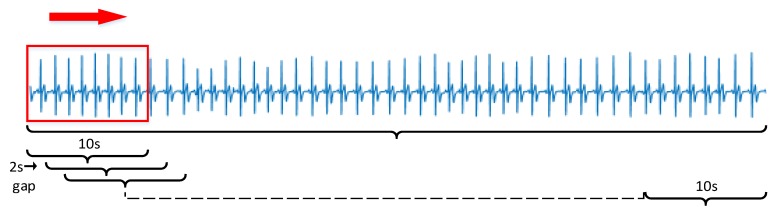
Data segment for ANN model.

To illustrate the effect of the distraction detection method in this paper, the more common and effective ANN model is selected for comparative experiments. ECG signals can be quantified in terms of time and frequency domains. Many studies have revealed that the time domain features are significantly related to drivers’ stress levels, while the frequency domain features are insignificantly related to the drivers’ stress levels [11]. In this paper, we extracted three times domain features (mean interbeat interval (mean IBI), standard deviation of IBIs (SDNN), and root mean squared difference of adjacent IBIs (RMSSD)), which have been frequently employed. Features were collected according to the following steps. First, for the ECG signal of the rest phase and normal driving 1 phase, consider the middle five minutes as the data for the low pressure and medium pressure states, and separately consider the middle 2.5 min of the ECG signal of numeral calculations 1 and numeral calculations 2 as the data for the high pressure state, which prevents the transition effects of different state transitions. Second, segment each part of the ECG signal, as illustrated in Figure 6. A moving window of 10 s with an overlap of 8 s was applied to each state. Third, use the pan tompkin algorithm coded in MATLAB to acquire the IBI data, and remove the abnormal points outside the normal range of IBIs (0.6 s–1.2 s). Last, calculate the three times domain features using Equations (1)–(3):

(1) $$Mean\;IBI=\;\frac{\sum_{i=1}^{n}IBI_{i}}{n},$$

where n = number of interbeat intervals, and $IBI_{i}$ = i-th interbeat interval.

(2) $$SDNN=\;\sqrt{\frac{\sum_{i=1}^{n}\left(IBI_{i}-\bar{IBI}\right)}{n-1}},$$

where $\bar{IBI}$ = average of interbeat intervals.

(3) $$RMSSD=\;\sqrt{\frac{\sum_{i=1}^{n-1}{\left(IBI_{i+1}-IBI_{i}\right)}^{2}}{n-1}},$$

**Figure 7:**
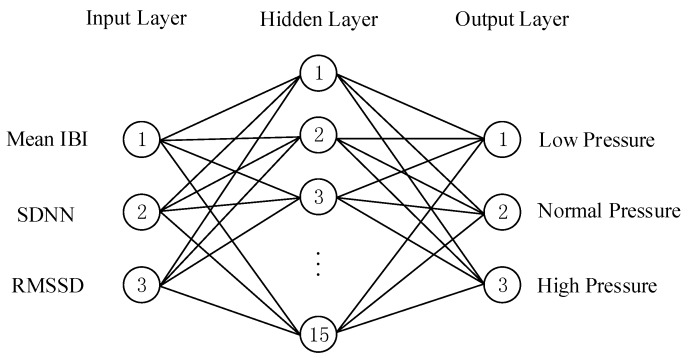
Three-layer ANN model structure.

The ANN model consists of three layers (input layer, hidden layer, and output layer), as shown in Figure 7. The input layer has three nodes for the three ECG features (mean IBI, SDNN, and RMSSD). The hidden layer has 15 neurons and uses a sigmoid activation function. The output layer has three nodes for the three types of driver status (low cognitive pressure, normal cognitive pressure, and high cognitive pressure).

We use a standard feed-forward and backpropagation neural network in this paper. A hyperbolic tangent sigmoid transfer function is applied as the transfer function of the hidden layer. A linear transfer function is used for the transfer function of the output layer. The scaled conjugate gradient is utilized as a backpropagation network learning function. Last, the data sets are randomly divided into a learning set and a test set: 70% of the data for learning the ANN model, 15% of the data for validation, and the remaining data for testing.

## 3. Results

### 3.1. Experimental Design Validation

First, we verified the validation of the experimental design. In our experiment, we increased the driver’s cognitive stress by adding the numerical calculations during normal driving, and we collected the ECG signals from which we extracted inter-beat intervals to reveal the change of the driver’s cognitive stress. A repeated measures ANOVA test of the inter-beat intervals of different period revealed that the inter-beat intervals were significantly (p < 0.001) altered by the cognitive stress level, which means that the driver’s physiological signals did change with the cognitive stress level in our experiment. Besides, we also performed a one-factor (cognitive stress level) within-subject ANOVA test of the ANN features, and the result revealed that the three time domain features (mean IBI (p < 0.001); SDNN (p < 0.001); RMSSD (p < 0.001)) were significantly different among the different cognitive stress levels at α = 0.001.

### 3.2. Classification Performance

**Figure 8:**
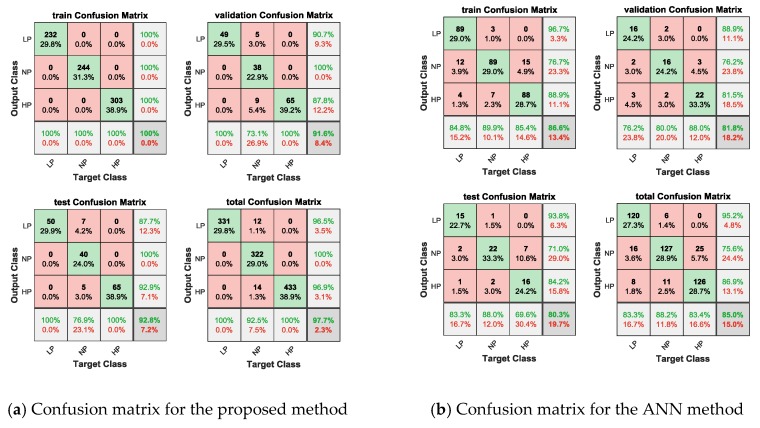
Confusion matrix for the proposed method and ANN method.

The classification accuracy of the proposed method was satisfactory for the learning set, validation set and test set. The input was the data set created in Section 2.4, and the outputs were low pressure (LP), normal pressure (NP), and high pressure (HP). The classification accuracies for the training set, validation set and test set were 100%, 91.6% and 92.8%, respectively, with no systematic bias in the sensitivity (true positive rate) and specificity (true negative rate), as shown in Figure 8a. The classification accuracy of the ANN method was also satisfactory for the learning set, validation set and test set. The input features were the mean IBI, SDNN, and RMSSD, and the outputs were low pressure (LP), normal pressure (NP), and high pressure (HP). The classification accuracies for the learning set, validation set and test set were 86.6%, 81.8% and 80.3%, respectively. As shown in Figure 8b, the sensitivity (true positive rate) and specificity (true negative rate) had no systematic bias in the learning, validation, and test sets.

**Figure 9:**
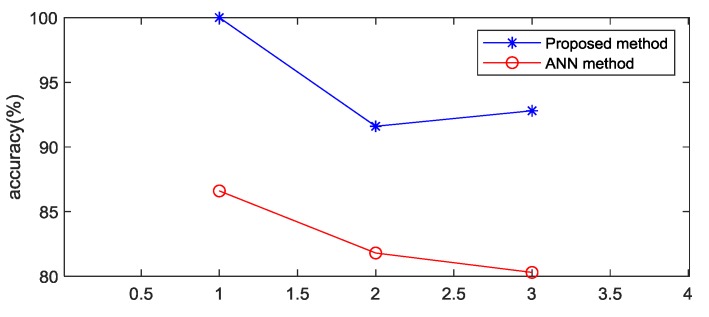
Comparison of the accuracies between the proposed method and the ANN method.

The results showed that the accuracies of the proposed method were substantially better than those of the ANN method (as shown in Figure 8 and Figure 9), which meant that the method proposed in this paper was feasible and performed better than the ANN method. Because the number of interbeat intervals in each picture was randomly selected, we discuss it in the next section.

### 3.3. Discussion of the Number of Inter-Beat Intervals in Each Picture

In this section, we discuss the number of interbeat intervals in each picture to determine the best number. Considering that the state of the driver can be represented when the segment of the ECG signal exceeds 10 s, we set the minimum number of IBIs in each image of the data set for the CNN model in the proposed method to 16 and increased the number by one each time. We employed the CNN model to train and test the data sets. To compare the performances of the proposed method on the data sets that consisted of the pictures of different number of IBIs, we repeatedly trained each data set 50 times and recorded the accuracy every time. We calculated the average and relative standard deviation (RSD) of the accuracies for each data set according to the following formula. The results are shown in Table 1.

(4) $$RSD=\;\frac{\sqrt{\frac{\sum_{i=1}^{50}\left(acc_{i}-\bar{acc}\right)}{50-1}}}{\bar{acc}},$$

where $acc_{i}$ = *i*-th accuracy in the 50 times, and $\bar{acc}$ = average of 50 accuracies.

**Figure 10:**
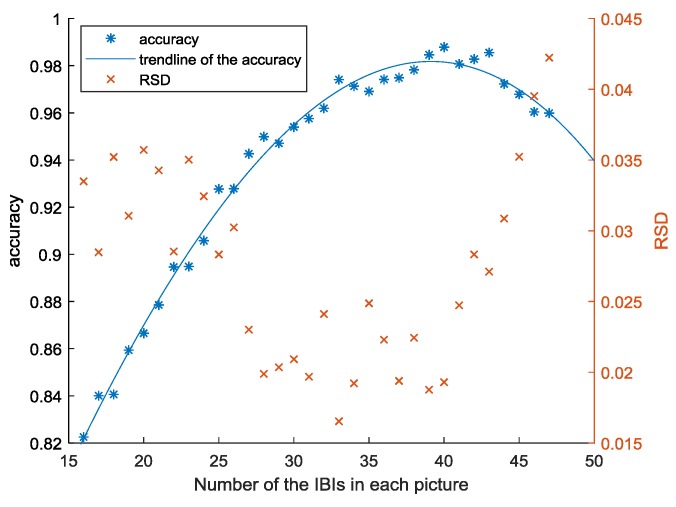
Performance of the CNN model for different data sets created with the pictures of different number of IBIs.

We drew a scatter plot to more intuitively analyze the data in the table. As shown in Figure 10, under the structure of the CNN model in Figure 5, the accuracy rate initially increases as the number of IBIs in each picture in the data set increases and shows a downward trend when the number exceeds 40. When the number is small, the information in a picture in the data set is limited and does not represent the status of low cognitive pressure, medium cognitive pressure or high cognitive pressure. Thus, the performance of the proposed method is poor, and the accuracy is relatively low. As the number increases, the amount of information in an image increases, and each image is increasingly more representative of a driver’s status. Thus, the performance of the proposed method improves, and the accuracy increases. However, when the number exceeds a certain number, which is 40 in this paper, the accuracy shows a downward trend. Because a picture contains a vast amount of information, the model can only learn the information of each state in the training set and cannot extend to that in the validation set and test set. Thus, the performance of the proposed method worsens on the validation set and the test set, and the accuracy decreases.

Based on the data acquired by our simulation experiment and the CNN model in Section 2.5, when the number of IBIs in each picture is 40, the accuracy of the proposed method is the highest and the relative standard deviation is at a relatively low level, as shown in Figure 10. The model performs steadily; therefore, we conclude that the best number of IBIs in a picture to classify a driver’s cognitive stress level is 40.

## 4. Discussion

In this paper, we proposed a novel method to detect and classify a drivers’ cognitive stress level by using a convolution neural network and achieved a satisfactory performance. First, based on the purpose of the experiment, we designed numeral calculations as the distracting driving task. The calculation difficulty was a two-digit addition and subtraction method that required carrying and borrowing. Second, we used the MP150-BioNomadix multichannel wireless physiological analysis recorder to obtain ECG signals of the driver for different cognitive stress levels in the driving simulation experiment. We also denoised the collected ECG signals by digital signal processing methods to accurately extract the interbeat intervals from the ECG signals. Third, we constructed the data set for the ANN model according to Figure 6 and the data set for the CNN model according to Figure 4. Fourth, we separately trained the ANN model and CNN model and compared the accuracies of the ANN method and the proposed method. The accuracies of the proposed method were substantially higher than the accuracies of the ANN method. Last, we compared the accuracies of the CNN model of the proposed method for different numbers of the interbeat intervals. 

The results of the paper show that the method is feasible and performs better than the methods based on the ANN model, which have been frequently applied in recent studies. Considering the feasibility and convenience of the method proposed in this paper, it can be applied in future research and provide a method for future driver monitoring. However, the method proposed has a weakness because it needs to take time to determine the best number of interbeat intervals contained in one picture for each driver. Although the method can achieve a good performance, the step of selecting the best number is inevitable.

Some future researches are needed to enhance the applicability of the proposed method. First, we just discussed the influence of the number of inter-beat intervals each picture contained, and the number of divided intervals was also needed to be discussed. Second, a real driving data is needed to validate the result of the paper because the experiment in this paper was performed in a driving simulator in which the conditions and the environment were controlled.

## Figures and Tables

**Table 1:** Performance on the CNN model for different data sets created with the pictures of different number of IBIs.

Number of the IBIs	$\bar{acc}$	RSD	Number of the IBIs	$\bar{acc}$	RSD
16	0.822538	0.033492962	32	0.961929	0.024117106
17	0.840061	0.028479318	33	0.974084	0.016536709
18	0.840612	0.035208451	34	0.97129	0.019219709
19	0.859388	0.031058617	35	0.969121	0.024874458
20	0.866564	0.035717147	36	0.974202	0.02229789
21	0.878523	0.034267829	37	0.974837	0.019388527
22	0.894613	0.028537217	38	0.978224	0.022435945
23	0.894845	0.035023254	39	0.98462	0.018765861
24	0.905776	0.032437347	40	0.987881	0.019297169
25	0.927712	0.028323002	41	0.980731	0.024735957
26	0.927837	0.030237982	42	0.982809	0.028324661
27	0.942642	0.023011742	43	0.985552	0.027109379
28	0.94991	0.019888319	44	0.972256	0.030862739
29	0.947111	0.020351297	45	0.967864	0.03522804
30	0.954013	0.020923643	46	0.960407	0.039515134
31	0.957572	0.019682664	47	0.959864	0.042232649

## References

[1] National Center for Statistics and Analysis (2018). Distracted Driving 2016.

[2] Ratcliff R., Strayer D. (2014). Modeling simple driving tasks with a one-boundary diffusion model. Psychon. Bull. Rev..

[3] Cooper J.M., Medeiros-Ward N., Strayer D.L. (2013). The impact of eye movements and cognitive workload on lateral position variability in driving. Hum. Factors.

[4] Reimer B., Mehler B., Wang Y., Coughlin J.F. (2012). A field study on the impact of variations in short-term memory demands on drivers’ visual attention and driving performance across three age groups. Hum. Factors.

[5] Strayer D.L., Fisher D.L. (2016). SPIDER: A framework for understanding driver distraction. Hum. Factors.

[6] National Center for Statistics and Analysis (2017). Distracted Driving 2015.

[7] Chen Z., Wu C., Zhong M., Lyu N., Huang Z. (2015). Identification of common features of vehicle motion under drowsy/distracted driving: A case study in Wuhan, China. Accid. Anal. Prev..

[8] Abouelnaga Y., Eraqi H.M., Moustafa M.N. (2017). Real-time distracted driver posture classification. arXiv.

[9] Mbouna R.O., Kong S.G., Chun M.G. (2013). Visual analysis of eye state and head pose for driver alertness monitoring. IEEE Trans. Intell. Transp. Syst..

[10] Craye C., Karray F. (2015). Driver distraction detection and recognition using RGB-D sensor. arXiv.

[11] Tjolleng A., Jung K., Hong W., Lee W., Lee B., You H., Son J., Park S. (2017). Classification of a Driver’s cognitive workload levels using artificial neural network on ECG signals. Appl. Ergon..

[12] Guardiola S., Girbés V., Armesto L., Dols J., Tornero J. (2017). Physiological Signal Analysis for Driver Stress Detection. https://www.researchgate.net/publication/320244776_PHYSIOLOGICAL_SIGNAL_ANALYSIS_FOR_DRDRIV_STRESS_DETECTION.

[13] Heikoop D.D., de Winter J.C.F., van Arem B., Stanton A.N. (2019). Acclimatizing to automation: Driver workload and stress during partially automated car following in real traffic. Transp. Res..

[14] Castaldo R., Melillo P., Bracale U., Caserta M., Triassi M., Pecchia L. (2015). Acute mental stress assessment via short term HRV analysis in healthy adults: A systematic review with meta-analysis. Biomed. Signal. Process. Control..

[15] Balasubramanian V., Bhardwaj R. (2018). Can cECG be an unobtrusive surrogate to determine cognitive state of driver?. Transp. Res..

[16] Almahasneh H., Chooi W.T., Kamel N., Malik A.S. (2014). Deep in thought while driving: An EEG study on drivers’ cognitive distraction. Transp. Res..

[17] Chai R., Naik G.R., Nguyen T.N., Ling S.H., Tran Y., Craig A., Nguyen H.T. (2016). Driver fatigue classification with independent component by entropy rate bound minimization analysis in an EEG-based system. IEEE J. Biomed. Health Inf..

[18] Yang L., Guan W., Ma R., Li X. (2019). Comparison among driving state prediction models for car-following condition based on EEG and driving features. Accid. Anal. Prev..

[19] Di Flumeri G., Borghini G., Aricò P., Colosimo A., Pozzi S., Bonelli S., Golfetti A., Kong W., Babiloni F. (2015). On the use of cognitive neurometric indexes in aeronautic and air traffic management environments. Proceedings of the International Workshop on Symbiotic Interaction.

[20] Di Flumeri G., Borghini G., Aricò P., Sciaraffa N., Lanzi P., Pozzi S., Vignali V., Lantieri C., Bichicchi A., Simone A. (2018). EEG-based mental workload neurometric to evaluate the impact of different traffic and road conditions in real driving settings. Front. Hum. Neurosci..

[21] Di Flumeri G., Borghini G., Aricò P., Sciaraffa N., Lanzi P., Pozzi S., Vignali V., Lantieri C., Bichicchi A., Simone A. (2019). EEG-based mental workload assessment during real driving: A taxonomic tool for neuroergonomics in highly automated environments. Neuroergonomics.

[22] Singh R.R., Conjeti S., Banerjee R. (2013). A comparative evaluation of neural network classifiers for stress level analysis of automotive drivers using physiological signals. Biomed. Signal. Process. Control..

[23] Singh R.R., Conjeti S., Banerjee R. (2014). Assessment of driver stress from physiological signals collected under real-time semi-urban driving scenarios. Int. J. Comput. Intell. Syst..

[24] Rajendra V. (2018). Characterization and Identification of Distraction During Naturalistic Driving Using Wearable Non-Intrusive Physiological Measure of Galvanic Skin Responses. Master’s Thesis.

[25] Lee B.G., Chung W.Y. (2016). Wearable glove-type driver stress detection using a motion sensor. IEEE Trans. Intell. Transp. Syst..

[26] Zhang Y.F., Gao X.Y., Zhu J.Y., Zheng W.L., Lu B.L. A novel approach to driving fatigue detection using forehead EOG. Proceedings of the 2015 7th International IEEE/EMBS Conference on Neural Engineering (NER).

[27] Zheng W.L., Lu B.L. (2017). A multimodal approach to estimating vigilance using EEG and forehead EOG. J. Neural Eng..

[28] Cafasso A., Karlsson S. (2017). Automatic Detection of Saccadic Eye Movements Using EOG for Analysing Effects of Cognitive Distraction during Driving. Master’s Thesis.

[29] LeCun Y., Bengio Y., Hinton G. (2015). Deep learning. Nature.

[30] Goodfellow I., Bengio Y., Courville A. (2016). Deep Learning.

[31] Gu J., Wang Z., Kuen J., Ma L., Shahroudy A., Shuai B., Liu T., Wang X., Wang G., Cai J. (2018). Recent advances in convolutional neural networks. Pattern Recognit..

[32] Kasakawa S., Yamanishi T., Takahashi T., Ueno K., Kikuchi M., Nishimura H. (2016). Approaches of phase lag index to EEG signals in Alzheimer’s disease from complex network analysis. Innovation in Medicine and Healthcare 2015.

[33] Chai R., Ling S.H., San P.P., Naik G.R., Nguyen T.N., Tran Y., Craig A., Nguyen H.T. (2017). Improving EEG-based driver fatigue classification using sparse-deep belief networks. Front. Neurosci..

[34] Gao Z., Wang X., Yang Y., Mu C., Cai Q., Dang W., Zuo S. (2019). EEG-based spatio–temporal convolutional neural network for driver fatigue evaluation. IEEE Trans. Neural Netw. Learn. Syst..

[35] Pan J., Tompkins W.J. (1985). A real-time QRS detection algorithm. IEEE Trans. Biomed. Eng..

